# Disturbance of serum lipid metabolites and potential biomarkers in the Bleomycin model of pulmonary fibrosis in young mice

**DOI:** 10.1186/s12890-022-01972-6

**Published:** 2022-05-04

**Authors:** Xiao-hui Yang, Fang-fang Wang, Xiao-sa Chi, Xiao-meng Wang, Jin-peng Cong, Yi Hu, Yu-zhu Zhang

**Affiliations:** 1 Department of Respiratory and Critical Care Medicine, Affiliated Hospital of Qingdao University, Qingdao, People’s Republic of China; 2 Department of Geriatrics, Affiliated Hospital of Qingdao University, Qingdao, 266000 People’s Republic of China

**Keywords:** Pulmonary fibrosis, Metabolites, Biomarkers, Mice

## Abstract

**Background:**

Altered metabolic pathways have recently been considered as potential drivers of idiopathic pulmonary fibrosis (IPF) for the study of drug therapeutic targets. However, our understanding of the metabolite profile during IPF formation is lacking.

**Methods:**

To comprehensively characterize the metabolic disorders of IPF, a mouse IPF model was constructed by intratracheal injection of bleomycin into C57BL/6J male mice, and lung tissues from IPF mice at 7 days, 14 days, and controls were analyzed by pathology, immunohistochemistry, and Western Blots. Meanwhile, serum metabolite detections were conducted in IPF mice using LC–ESI–MS/MS, KEGG metabolic pathway analysis was applied to the differential metabolites, and biomarkers were screened using machine learning algorithms.

**Results:**

We analyzed the levels of 1465 metabolites and found that more than one-third of the metabolites were altered during IPF formation. There were 504 and 565 metabolites that differed between M7 and M14 and controls, respectively, while 201 differential metabolites were found between M7 and M14. In IPF mouse sera, about 80% of differential metabolite expression was downregulated. Lipids accounted for more than 80% of the differential metabolite species with down-regulated expression. The KEGG pathway enrichment analysis of differential metabolites was mainly enriched to pathways such as the metabolism of glycerolipids and glycerophospholipids. Eight metabolites were screened by a machine learning random forest model, and receiver operating characteristic curves (ROC) assessed them as ideal diagnostic tools.

**Conclusions:**

In conclusion, we have identified disturbances in serum lipid metabolism associated with the formation of pulmonary fibrosis, contributing to the understanding of the pathogenesis of pulmonary fibrosis.

**Supplementary Information:**

The online version contains supplementary material available at 10.1186/s12890-022-01972-6.

## Background

Idiopathic pulmonary fibrosis (IPF) is a chronic, progressive, and destructive interstitial lung disease, which is a fibroproliferative disease with a clinical course similar to that of many malignant tumors and can be described as a "cancer that is not cancer" [1]. In recent years, the incidence of pulmonary fibrosis (PF) has been on the rise and has become a worldwide health problem [2]. Most cases are diagnosed in patients over 60 years of age and patients have an extremely poor prognosis, with a median survival of only 3–5 years if treatment is missing [3]. Early diagnosis is difficult due to the absence of specific clinical manifestations. 5-year survival in IPF is worse than in several types of cancer (e.g., breast, ovarian, and colorectal), and most patients die from clinical progressive respiratory failure [4, 5].

The main pathological features of PF are proliferation and differentiation of fibroblasts, abnormal deposition of large amounts of extracellular matrix (ECM), infiltration of inflammatory cells, and destruction of alveolar structures. It is currently believed that the development of pulmonary fibrosis can be divided into two stages, namely the early stage of alveolar inflammation and the later stage of hyperfibrotic repair [6, 7]. In the early stage of alveolar inflammation, activated mesenchymal and infiltrating cells in the lung secrete a series of cytokines, including tumor necrosis factor-alpha (TNF-ɑ), chemokine (CXC), and cell adhesion cytokine (CAM), which interact to promote further aggregation of inflammatory mononuclear cells. In the late stages of fibrosis, inflammatory cells and mesenchymal cells (fibroblasts and myofibroblasts) are activated to participate in the repair and remodeling of lung tissues and blood vessels, ultimately leading to the development of pulmonary fibrosis [8–10]. In addition, transforming growth factor-β1 (TGF-β1) and α-smooth muscle actin (α-SMA) are key fibrogenic factors that play an important role in the differentiation and proliferation of fibroblasts to myofibroblasts [11, 12]. Currently, there is a lack of effective methods to prevent or reverse the pathological process of pulmonary fibrosis.

The most frequently used animal model of IPF is bleomycin-induced lung injury and fibrosis in mice, although it does not fully recapitulate the pathology of the disease [13]. Bleomycin is a chemotherapeutic agent that causes epithelial cell death in the first three days after dosing, followed by excessive inflammatory infiltration around the first week and finally fibroblast activation, ECM deposition, and fibrosis around the second week after injury [14, 15]. To date, most of the metabolomic studies done using the bleomycin-induced mouse IPF model have focused on the lung tissue of mice as well as the feces in the intestine, and very little research has been done on their plasma [16, 17]. Although many investigators have used plasma or lung tissue from patients with IPF for their studies, most of the patients used for these studies were in the late stages of IPF due to the difficulty of early diagnosis of IPF [18, 19]. Thus, our lack of understanding of the early stages of IPF onset does not facilitate the development of targeted drugs to slow the progression of IPF.

Since IPF shows significant heterogeneity in its natural course and prognosis, biomarkers can play an auxiliary role in disease diagnosis, disease assessment, treatment response, and disease risk prediction in pulmonary fibrosis based on clinical, imaging, and pathology [20, 21]. Currently, the more studied IPF biomarkers are mainly divided into genes or protein-based markers associated with alveolar epithelial cell injury, fibroplasia, extracellular matrix remodeling, and immune dysfunction and their combined use are of great value in improving the accuracy of IPF diagnosis, disease assessment, and judgment of prognosis [22, 23]. However, most IPF biomarkers lack specificity and reproducibility and thus are not widely accepted [24]. As a novel histological technique, metabolomics is often used to find biomarkers with high sensitivity and specificity and good reproducibility that are applied for early diagnosis, guiding personalized treatment, and predicting the risk of disease progression and death.

IPF is considered to be a metabolism-related disease. During pulmonary fibrosis, the activation of fibroblasts depends to some extent on glycolysis, and the metabolic reprogramming associated with glycolysis can influence the severity of IPF [25]. In addition, the activation and distribution of M2 macrophages in fibrotic alveoli are dependent on glycolysis [26]. Analysis of IPF tissue by mass spectrometry identified a variety of metabolites associated with pulmonary fibrosis, mainly involving the adenosine triphosphate degradation pathway and the glycolytic pathway [19]. Feng Yan et al. identified 62 unique lipids in the plasma of IPF patients, including 24 kinds of glycerophospholipids (GP), 30 kinds of glycerolipids (GL), 3 kinds of sterol lipids, 4 kinds of sphingolipids, and 1 kind of fatty acids [18]. Kang YP et al. assay of IPF lung tissue revealed 25 metabolites of IPF, mainly involving the adenosine triphosphate degradation pathway, glycolytic pathway, glutathione biosynthesis pathway, and ornithine aminotransferase pathway [19]. This suggests that metabolites give us new perspectives to explore the pathogenesis of IPF [27].

In this paper, we constructed the mouse model of IPF with bleomycin from the perspective of metabolomics and attempted to find specific metabolites for different stages of IPF from the perspective of metabolites for pulmonary fibrosis diagnosis and disease assessment.

## Methods

### Animal, fibrotic models, and sample collection

Specific-pathogen-free C57BL/6J male mice (about 8 weeks of age) were purchased from Beijing Vital River Laboratory Animal Technology Co., Ltd. All mice were housed separately in individually ventilated cages at 22–25 °C, the humidity of 46–65% and 12-h day/night cycle, and fed a normal chow diet. They were randomly assigned to the three groups (Control, M7, and M14) with 10 mice in each group. All experiments were approved by the Ethics Committee of Affiliated Hospital of Qingdao University (Ethics No. QYFYW2LL26275), and all methods were carried out following relevant guidelines and regulations. This study was carried out in compliance with the ARRIVE guidelines.

Mice were first properly anesthetized with 1% sodium pentobarbital and then intratracheally instilled with Bleomycin or saline (NaCl) on day 0. Mice were instilled using 2.0 mg/kg of Bleomycin in saline solution for model mice. Control mice received saline solution only. Lung tissues and sera from mice were collected for histopathological, immunohistochemistry, and metabolomic assays from the drug-induced mice on the 7th day, and 14th day, as well as from control mice (0th day). Blood is collected from mice employing orbital plexus blood collection.

### Pathological analysis, immunohistochemistry and western blots

Mice were deeply anesthetized with sodium pentobarbital and died from blood loss after incision of the abdominal aorta. After induction of pulmonary fibrosis, the heart was perfused with PBS and the left lobe was injected with approximately 250 μl 4% paraformaldehyde, and then the 4% paraformaldehyde-fixed tissue was routinely processed, embedded in paraffin, and sectioned. Two adjacent slices were stained with TGF-β1, HE, and Masson, respectively, and the stained slides were scanned with PANORAMIC DESK/MIDI/250/1000 (3DHISTECH, Hungary), and representative slice areas are shown in the screenshots. Subsequently, the software's image analysis system was used to read and analyze the intensity of positivity of the measured areas.

The expression of TGF-β1 and ɑ-SMA in lung tissue was determined by the Western Blot technique, and the grayscale values of the target bands in the Western Blot results were analyzed using Alpha software (Alpha Innotech, US). Briefly, tissues were homogenized by lysis buffer (0.22 M Tris–HCl (pH 6.8), 8.8% SDS, 44.4% glycerol) and centrifuged at 12,000×g for 10 min at 4 °C, and the supernatant was collected. After sample staining, equal amounts of proteins were loaded in each well and subjected to SDS-PAGE gel electrophoresis. Proteins were transferred to nitrocellulose membranes and detected with primary antibodies (Santa Cruz Biotech, US). Subsequently, detection and signal visualization were performed using appropriate horseradish peroxidase-coupled secondary antibodies (Santa Cruz Biotech, US) and enhanced chemiluminescence reagents (GE Healthcare UK).

### UPLC-MS metabolome profiling

Metabolite extracts were obtained from serum samples following methanol-assisted protein precipitation and then analyzed using an LC–ESI–MS/MS system (UPLC, ExionLC AD, https://sciex.com.cn/; MS, QTRAP® System, https://sciex.com/). Liquid phase separation was performed using a Waters ACQUITY UPLC HSS T3 C18 column (1.8 µm, 2.1 mm × 100 mm). LIT and triple quadrupole (QQQ) scans were acquired on a triple quadrupole linear ion trap mass spectrometer (QTRAP) controlled by Analyst 1.6.3 software (Sciex) QTRAP® LC–MS/MS system. A specific set of MRM shifts was monitored for each period based on the metabolite elution in each period. Software Analyst 1.6.3 was used to process the mass spectrometry data. Several existing mass spectrometry public databases were consulted for metabolite structure analysis, mainly massbank (http://www.massbank.jp/), knapsack (http://kanaya.naist.jp/knapsack/), HMDB (http://www.hmdb.ca/) and Metlin (http://metlin.scripps.edu/index.php). Qualitative analysis was performed based on retention times and mass-to-charge ratios of parent and daughter ions of test substances from internal and other public databases. The quantitative analysis was performed by the triple quadrupole mass spectrometry multiple reaction monitoring (MRM) methods. The signal intensity of the characteristic ions is obtained in the detector. MultiQuant is used to integrate and calibrate the chromatographic peaks. The peak area of each chromatographic peak represents the relative amount of the corresponding substance [28, 29].

### Data analysis

At first, the homogeneity and reproducibility of metabolite data were visualized by Uniform Manifold Approximation and Projection (UMAP) and principal component analysis (PCA), respectively. Then, orthogonal partial least squares discriminant analysis (OPLS-DA) was further applied to obtain the variable importance (VIP) values for each metabolite to measure the contribution of the variables to the model. The validity of the OPLS-DA model is determined by R2Y (interpretability of the model for the categorical variable Y) and Q2 (predictability of the model).

Along with the multivariate statistical methods, Student t-tests were used to measure the significance of each metabolite. The statistically significantly changed metabolites were selected using the criteria of p-value less than 0.05 and absolute log2 Foldchange (log2 FC) larger than 0.58. Heatmap and hierarchical clustering analysis were performed using the Heatmap package in R software (version 4.0.5). Machine learning can be employed to reduce the dimensionality of metabolomic datasets and improve the accuracy of predictions. The random forest algorithm (package varSelRF) in machine learning is applied to select the characteristic material, and its performance is subsequently evaluated using the receiver operating characteristic curve (ROC). The ggplot2 package in R software for data visualization.

The analysis of metabolic pathways was performed by the Kyoto Encyclopedia of Genes and Genomes (KEGG) (https://www.kegg.jp/) and the Metaboanalyst (https://www.metaboanalyst.ca/MetaboAnalyst/), and the results were shown using bubble plots [30].

## Results

### The establishment of pulmonary fibrosis mouse models induced by bleomycin

**Fig. 1:**
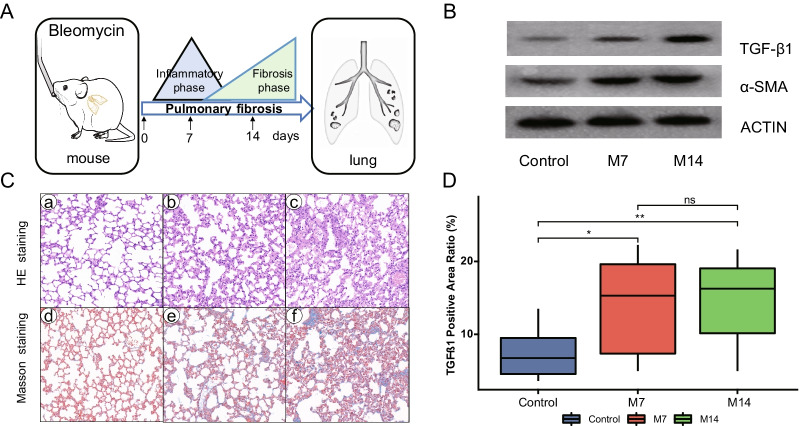
Bleomycin mouse models. **A** Study design, intratracheal instillation of bleomycin or saline (NaCl) was administered to anesthetized mice at day 0. Lung tissues and serum were collected from mice at 7 and 14 days of bleomycin induction and from control mice for histopathological and metabolomic assays.; **B** TGF-β1 and ɑ-SMA expression of mouse lung tissues were analyzed using western blot at 7 and 14 days after bleomycin instillation; **C** Lung histopathology after a single dose of bleomycin treatment (HE and Masson staining). Representative hematoxylin- and eosin-stained (a, b, and c) and Masson's trichrome-stained (d, e, and f) lung tissue sections are shown. The mice were sacrificed at 7 (b and e) and 14 (c and f) days after bleomycin instillation. Except for the PBS control (a and d), all panels showed lung sections from bleomycin-treated mice. The lungs were inflated with 10% buffered formaldehyde. Original magnification, 20X; **D** The results of TGF-β1 immunohistochemistry. Positive Area Ratio, reflecting the proportion of the positive area. Statistical significance by two-tailed Student’s t-tests: ^∗^p < 0.05; ^∗∗^p < 0.01; ns, not significant

To gain a comprehensive understanding of the metabolomic changes during the formation of pulmonary fibrosis, we injected saline solution (NaCl) containing 2.0 mg/kg bleomycin into the trachea of mice (Fig. 1A). No significant changes in body weight and dietary intake were observed in bleomycin-induced mice compared to the control group (p > 0.05). Lung tissue and serum were collected from 10 mice on day 7 (M7) and day 14 (M14) according to the stage of pulmonary fibrosis. TGF-β1 is a key cytokine in the process of lung fibrosis, and the results of western blot (Fig. 1B, Additional file 1: Figure S1A, and Additional file 4: Figure S4) and immunohistochemistry (Fig. 1D) of TGF-β1 in lung tissues showed that the level of TGF-β1 increased significantly with the increase of fibrosis (p < 0.05). In addition, α-SMA is a cellular marker for fibrosis, whereas the results of western blot of lung tissue showed no significant change in the expression level of α-SMA (Fig. 1B and Additional file 1: Figure S1B) (p > 0.05). The results from HE and Masson staining showed that the lung tissue showed an increase in collagen fibers closely associated with pulmonary fibrosis (Fig. 1C).

### The altered metabolism in serum of mouse fibrosis models

**Fig. 2:**
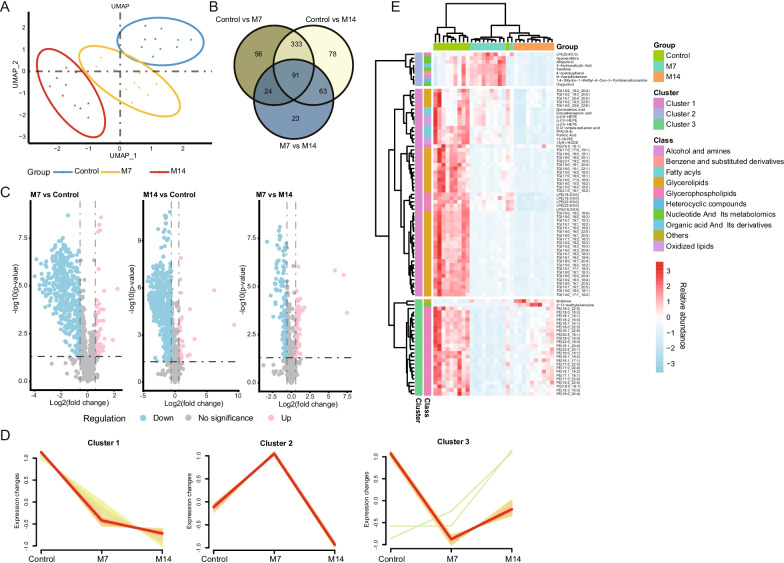
Metabolome profiling of serum. **A** UMAP multivariate statistical model of the Control, M7, and M14 groups; **B** Venn diagram analysis of the differential metabolites, with different colored circles representing the number of differential metabolites between groups. upward trend; **C** Volcano plot of metabolites, with blue representing downward trend, gray representing insignificance, and red representing upward trend; **D** Clustering diagram of 91 metabolites that varied significantly among Control, M7, and M14 groups; **E** Heatmap of the relative abundance of representative differential serum metabolites among Control, M7, and M14 groups. GP, glycerophospholipids; GL, glycerolipids; FA, fatty acyls.

The time-series metabolic profile in the serum of the mouse fibrosis models was obtained using mass spectrometry (MS) assays. A total of 1465 metabolites were detected in serum samples, which can be classified into 19 types, mainly GP, GL, and fatty acyl (FA). UMAP showed a clear trend of separation of metabolites between the groups, showing differences between them (Fig. 2A and Additional file 2: Figure S2A). Moreover, PCA and OPLS-DA analysis confirmed the above results (Additional file 2: Figure S2).

We performed univariate Student t-tests in each group and found that a total of 504 and 565 metabolites differed in M7 and M14, respectively, compared to the control group, while 201 differential metabolites were found between M7 and M14 (log2 FC > 0.58, p-value < 0.05) (Fig. 2B). In IPF mice sera, about 80% of differential metabolite expression was down-regulated, and only a small proportion was up-regulated (Fig. 2C and Additional file 3: Figure S3). Lipids (e.g., GL, GP) and Fatty acyl together accounted for more than 80% of the differential metabolites with down-regulated expression. In addition, many differential metabolites existed between M7 and M14. Subsequently, we found 91 metabolites that were all significantly altered between M7, M14, and Controls by Venn diagram analysis (Fig. 2B). Clustering of these differential metabolites above showed that they exhibited three trends of variation. The metabolites in Cluster 1 were mainly GL-like substances, which decreased significantly in the pulmonary fibrosis model while maintaining high concentrations in Control; the metabolites in Cluster 2 were mainly Nucleotide and its metabolomics-like substances, which increased significantly in the initial phase of the pulmonary fibrosis model and then decreased significantly, with higher expression levels in M7; in Cluster 3, except for Kojibiose and 2'-O-methyladenosine, the other metabolites were mainly GP-like substances, which decreased significantly in the initial stage of the pulmonary fibrosis model and then increased, presenting a lower expression level in M7 (Fig. 2D, E).

### KEGG pathway analysis and changes of enrichment pathway of pulmonary fibrosis in mice

**Fig. 3:**
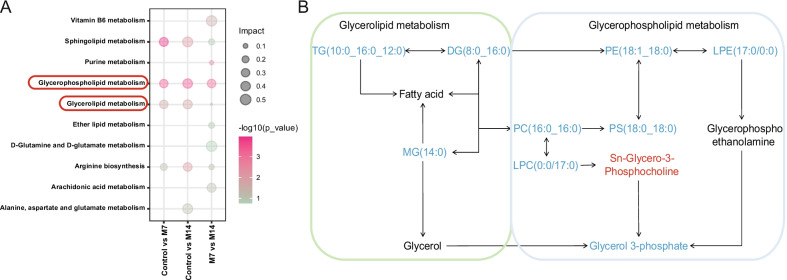
Pathway analysis of differential metabolites in the serum of mice with pulmonary fibrosis. **A** KEGG substance pathway analysis, the color of the dot represents p-value, and the redder the dot, the more significant the enrichment. The size of the point represents the pathway impact. The pathway impact is a weight calculation based on topology analysis, the larger the pathway impact is, the more plausible the corresponding pathway is. **B** Simplified scheme of Glycerolipid pathway and Glycerophospholipid pathway and the connection with them. Red, indicating upregulation of metabolites; blue, indicating downregulation of metabolites

The pathway analysis of differential metabolites during lung fibrosis in mice was performed based on KEGG. Pathway enrichment analysis of differential metabolites between IPF models (M7 and M14) and Controls was enriched for Sphingolipid metabolism, Glycerophospholipid metabolism, and Glycerolipid metabolism pathway (Fig. 3A). Interestingly, the vast majority of metabolites enriched in Glycerophospholipid metabolism, Glycerolipid metabolic pathway were downregulated (Fig. 3B). In addition, analysis of differential metabolites between M7 and M14 enriched pathways such as Vitamin B6 metabolism, D-glutamine, and D-glutamate metabolism, and Purine metabolism pathways.

### Screening for potential metabolic biomarkers of pulmonary fibrosis in mice

**Fig. 4:**
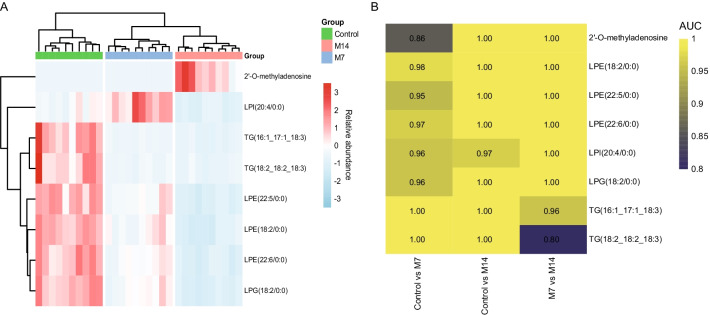
Heatmap of biomarkers for random forest screening with their AUC heatmap. **A** Heatmap of the relative abundance of the eight metabolites screened by random forest after normalization, with red representing high expression and blue representing low expression; **B** AUC heatmap of the eight metabolites

The above analysis indicates the presence of many metabolites in the serum that change with the degree of lung fibrosis in mice. Thus, a random forest machine learning model was developed to select appropriate metabolic markers of pulmonary fibrosis. With this model, eight metabolites were screened, which were 2'-O-methyladenosine, LPI (20:4/0:0), LPE (18:2/0:0), LPE (22:5/0:0), LPE (22:6/0:0), LPG (18:2/0:0), TG (16:1_17:1_18:3) & TG (18:2_18:2_18:3) (Fig. 4A). We then assessed the diagnostic value of these eight metabolites using ROC curves, and their area under the ROC curve (AUC) was greater than 0.8. This implies that the model is an ideal diagnostic tool for pulmonary fibrosis (Fig. 4B).

## Discussion

Here, we present an exhaustive characterization of metabolite changes during pulmonary fibrosis in mice based on metabolomics techniques. The 1465 metabolites in mouse serum were analyzed by mass spectrometry. Interestingly, more than one-third of these metabolites (504 and 565, respectively) showed a significant decrease in sera from bleomycin-treated mice, suggesting a strong impact of pulmonary fibrosis on mouse metabolism.

The altered metabolic pathways we observed in bleomycin-induced mouse sera are consistent with those reported for patients with IPF, indicating the reliability of the present model for IPF studies. Lipids play an irreplaceable role in cellular energy storage, structure, and signaling, and have been implicated in the development of several respiratory diseases, including cystic fibrosis, asthma, and chronic obstructive pulmonary disease (COPD) [31, 32]. However, the role that lipids play in IPF is not fully understood. In the plasma of patients with IPF, a decrease in GL and GP has been found [18]. In a mouse model of pulmonary fibrosis, we likewise observed changes in these lipids, involving disturbances in pathways such as glycerophospholipid metabolism and glycerol ester metabolism. In addition, Weckerle et al. found a reduction of more than half of the metabolites or lipids in the lung tissue of mice with pulmonary fibrosis also induced by bleomycin, which may be related to higher energy requirements, proliferation, tissue remodeling, collagen deposition, and inflammation [17]. Thus, it can be tentatively concluded that the development of pulmonary fibrosis is closely related to the disruption of metabolites such as lipids, which requires further study. Interestingly, our clustering analysis of differential metabolites revealed that metabolites in some clusters maintain a unique association with the process of pulmonary fibrosis. For example, GL-like metabolites in Cluster 1 showed reduced concentrations in the pulmonary fibrosis model, while Nucleotide and Its metabolomics-like substances in Cluster 2 showed increased and subsequently reduced expression at the beginning of pulmonary fibrosis, suggesting that such substances are likely to be involved in the inflammatory response during pulmonary fibrosis and play a unique role in the formation of pulmonary fibrosis. In addition, the high levels of Kojibiose and 2'-O-methyladenosine in M14 may predict the deepening of pulmonary fibrosis and need to be further investigated.

Fiber metabolism is an emerging and exciting avenue of research. It has been shown that metabolic reprogramming associated with glycolysis can influence the severity of IPF, while imbalances in lipid mediators (e.g. prostaglandins and lysophospholipids) can also drive the fibrotic response in the context of IPF [25, 33]. We have likewise observed perturbations with related lipid metabolites in IPF models, and pathways such as sphingolipid metabolism, glycerophospholipid metabolism, and glycerolipid metabolism may be involved in the inflammatory and fibrotic phases of pulmonary fibrosis, which may contribute to a deeper understanding of pulmonary fibrosis from the perspective of lipid metabolism.

Pulmonary fibrosis is regulated by a complex network of multiple cells and cytokines, of which persistent activation of fibroblasts/myofibroblasts, disruption of collagen fiber metabolism, and remodeling of the extracellular matrix are the pathological features [31, 34, 35]. TGF-β1 plays a key role in the inflammation, injury, and repair of pulmonary fibrotic disease. TGF-β1 stimulates fibroblast synthesis of ECM, leading to abnormal deposition of ECM in the lung and contributing to the conversion of lung fibroblasts into myofibroblasts [36]. α-SMA is a marker of activated fibroblasts and a key fibrogenic factor [12]. We observed abnormal deposition of extracellular matrix in the lung tissue and progressive fibrosis of the tissue at 7 and 14 days after bleomycin injection in the trachea of mice. The increased concentration of TGF-β1 in tissues also predicted a strong inflammatory response in lung tissue, which accelerated the process of tissue fibrosis. In addition, we did not observe a significant increase in the level of α-SMA in the tissues. The role played by α-SMA in the process of lung fibrosis needs to be studied to elaborate. Combined with previous studies, it is not difficult to find that intense inflammation, the proliferation of fibroblastic cells, and fibrosis in lung tissue are closely related.

Due to the relative difficulty in the diagnosis of IPF, attempts have been made to find various types of non-invasive biomarkers to be applied in the ancillary diagnosis of IPF, especially in serum. Generally, the possible biomarkers can be divided into three different groups according to the different pathways involved in the pathogenesis of IPF, namely biomarkers associated with alveolar epithelial cell dysfunction, biomarkers associated with extracellular matrix remodeling, and fibroproliferation, and biomarkers associated with immune dysfunction [22]. Studies from several large cohorts suggest that the MUC5B promoter SNP may be of great value in the diagnosis and prognostic assessment of IPF disease and may serve as an important biomarker for IPF [37, 38]. In addition, protein-based markers such as mucin 5B (MUC5B) and matrix metalloproteinase 7 (MMP7) are promising for consideration in clinical practice [22]. Interestingly, lysophosphatidylcholine (LysoPC) was found to have potential as a biomarker in the sera of IPF patients [27]. To further explore the clinical value of these altered metabolites, we analyzed 91 significantly altered metabolites using a random forest algorithm and screened for 8 biomarkers. Our analysis of these 8 metabolites by ROC curves also showed extremely high diagnostic performance as potential biomarkers for subsequent pulmonary fibrosis diagnosis and disease assessment.

## Conclusions

In conclusion, we found that the formation of IPF was accompanied by dramatic metabolic disturbances in the serum, with downregulation of a large number of lipid metabolites and abnormal fluctuations in pathways such as glycerolipid metabolism and glycerophospholipid metabolism. Increased concentrations of TGF-β1 and α-SMA in lung tissue were also associated with a deepening degree of IPF. In addition, several metabolites we screened from serum are expected to serve as potential biomarkers for clinical studies.

## Supplementary Information

**Additional file 1:** Results of western blot analysis of TGF-β1 and ɑ-SMA in lung tissue.

**Additional file 2:** Statistical analysis of metabolomic data by different methods.

**Additional file 3:** Composition of metabolites differing between groups in the sera of mice with pulmonary fibrosis.

**Additional file 4:** Original pictures of TGF-β1 and ɑ-SMA expression in mouse lung tissue were analyzed by Western blot at 7 and 14 days after bleomycin perfusion.

**Additional file 5:** The 91 metabolites that differed significantly among Control, M7 and M14.

## Data Availability

The raw data of metabolome datasets can be obtained from iProX database (accession number: IPX0004172000).

## Abbreviations

IPF: Idiopathic pulmonary fibrosis
PF: Pulmonary fibrosis
ROC: Receiver operating characteristic curves
ECM: Extracellular matrix
TNF-ɑ: Tumor necrosis factor-alpha
CXC: Chemokine
CAM: Cell adhesion cytokine
MRM: Multiple reaction monitoring
UMAP: Uniform Manifold Approximation and Projection
PCA: Principal component analysis
OPLS-DA: Orthogonal partial least squares discriminant analysis
VIP: Variable importance
log2 FC: Log2 Foldchange
KEGG: The Kyoto Encyclopedia of Genes and Genomes
MS: Mass spectrometry
GP: Glycerophospholipids
GL: Glycerolipids
FA: Fatty acyl
AUC: Area under the ROC curve
COPD: Chronic obstructive pulmonary disease
MUC5B: Mucin 5B
MMP7: Matrix metalloproteinase 7
LysoPC: Lysophosphatidylcholine
