# Supplementary material for: Disturbance of serum lipid metabolites and potential biomarkers in the Bleomycin model of pulmonary fibrosis in young mice
Source: BMC Pulm Med. 2022 May 4;22:176. doi: 10.1186/s12890-022-01972-6 (PMC9066762; doi:10.1186/s12890-022-01972-6)
Supplement: Supplementary file 1 — Additional file 1: Results of western blot analysis of TGF-β1 and ɑ-SMA in lung tissue. [file 12890_2022_1972_MOESM1_ESM.pdf]

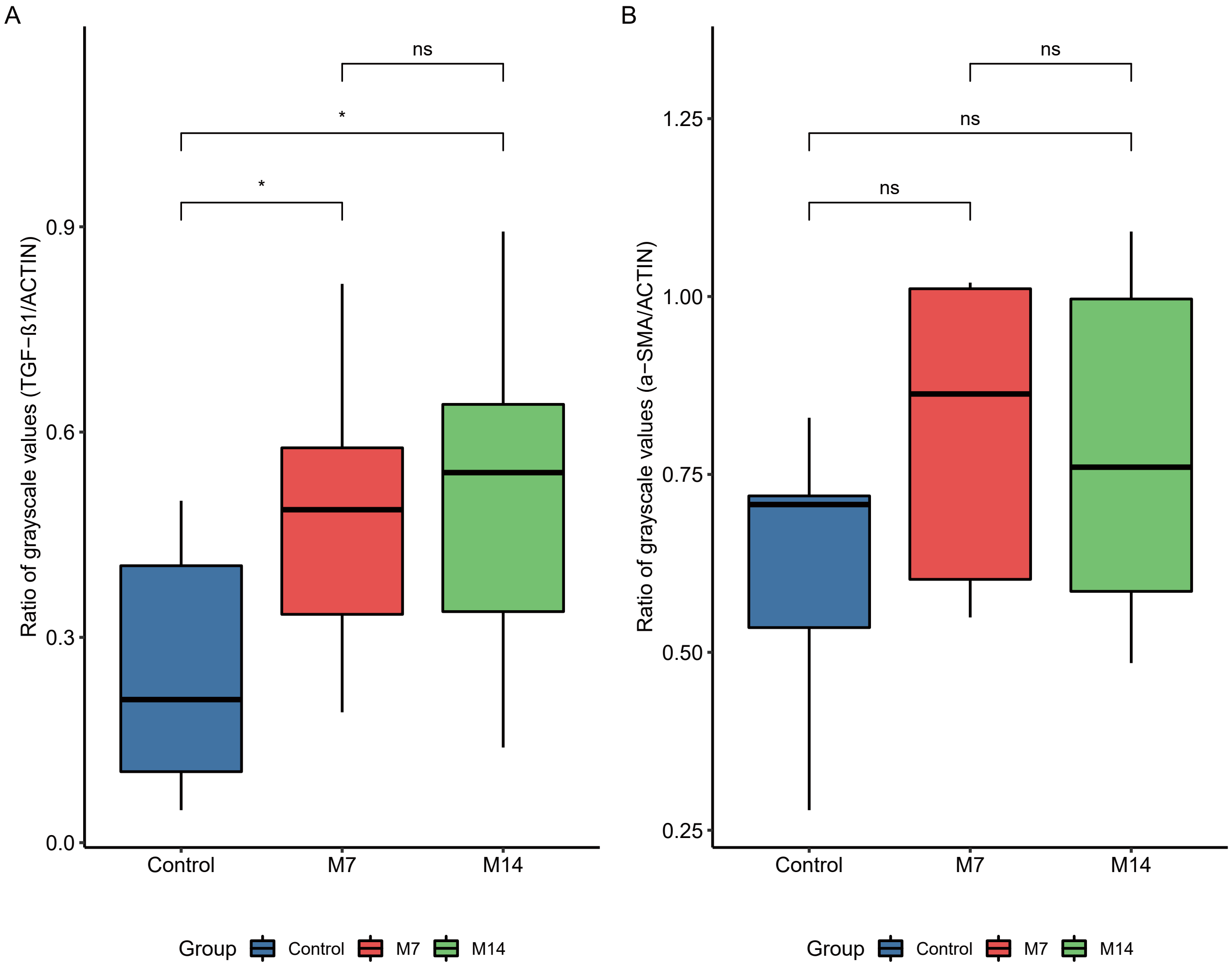

Figure S1 Results of western blot analysis of TGF-β1 and α-SMA in lung tissue

(A) Western blot of TGF-β1 in lung tissue of mice at 7 and 14 days after bleomycin perfusion, the ratio of grayscale values of TGF-β1 to internal reference bands (ACTIN); (B) Western blot of α-SMA in lung tissue of mice at 7 and 14 days after bleomycin perfusion, the ratio of grayscale values of TGF-β1 to internal reference bands (ACTIN). Statistical significance by two-tailed Student's t-tests: * $p < 0.05$; ns, not significant.
