# Supplementary material for: Disturbance of serum lipid metabolites and potential biomarkers in the Bleomycin model of pulmonary fibrosis in young mice
Source: BMC Pulm Med. 2022 May 4;22:176. doi: 10.1186/s12890-022-01972-6 (PMC9066762; doi:10.1186/s12890-022-01972-6)
Supplement: Supplementary file 2 — Additional file 2: Statistical analysis of metabolomic data by different methods. [file 12890_2022_1972_MOESM2_ESM.pdf]

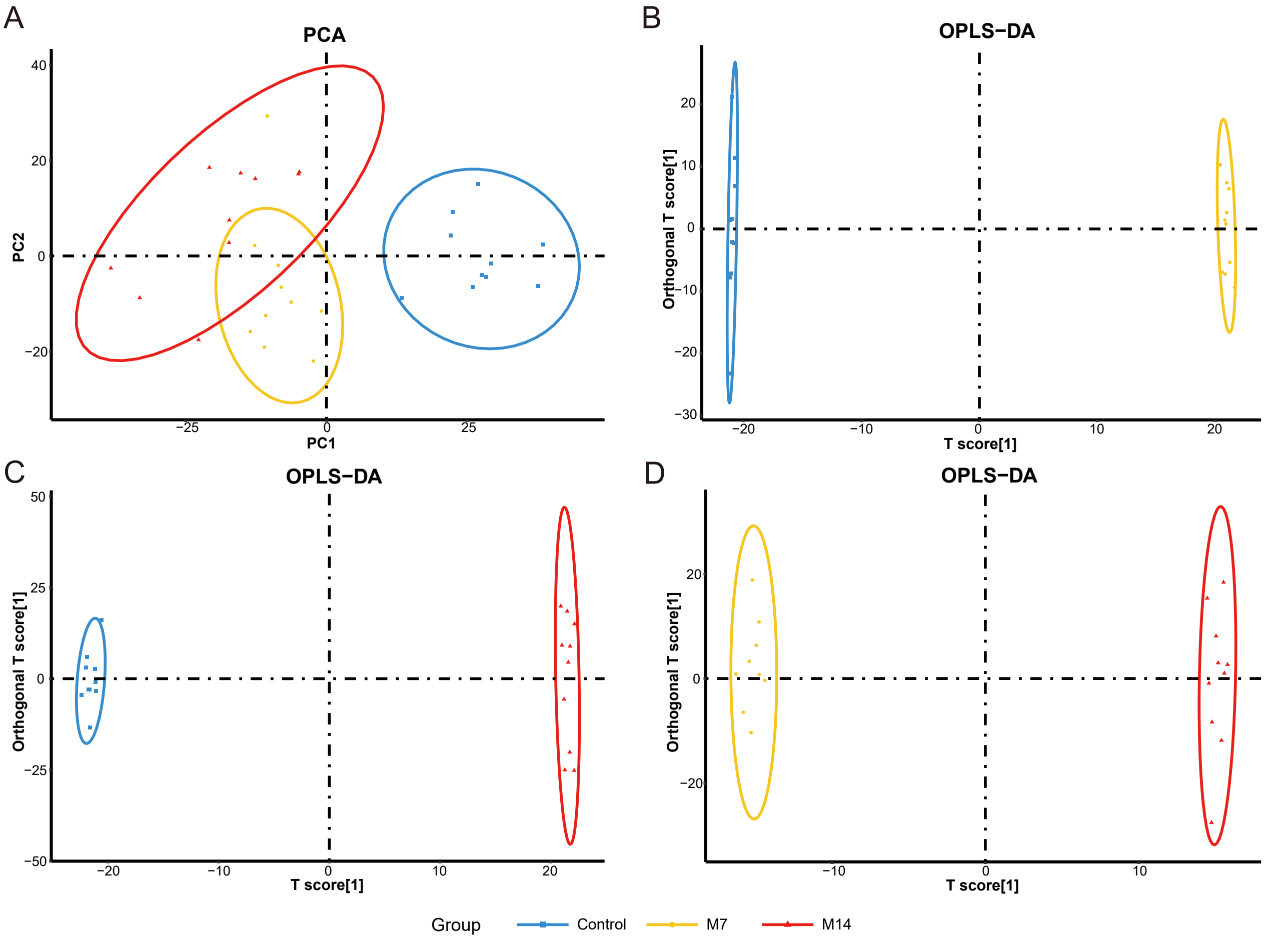

Figure S2 Statistical analysis of metabolomic data by different methods

(A) Principal component analysis (PCA) of Control, M7 and M14 (R2X=0.585, Q2=0.371); (B-D) The clustering analyses of orthogonal partial least-squares discriminant analysis (OPLS-DA) of Control and M7 group (R2X=0.614, R2Y=1, Q2=0.944), Control and M14 group (R2X=0.59, R2Y=1, Q2=0.928), and M7 and M14 group (R2X=0.491, R2Y=0.999, Q2=0.885).
