# Supplementary material for: Disturbance of serum lipid metabolites and potential biomarkers in the Bleomycin model of pulmonary fibrosis in young mice
Source: BMC Pulm Med. 2022 May 4;22:176. doi: 10.1186/s12890-022-01972-6 (PMC9066762; doi:10.1186/s12890-022-01972-6)
Supplement: Supplementary file 3 — Additional file 3: Composition of metabolites differing between groups in the sera of mice with pulmonary fibrosis. [file 12890_2022_1972_MOESM3_ESM.pdf]

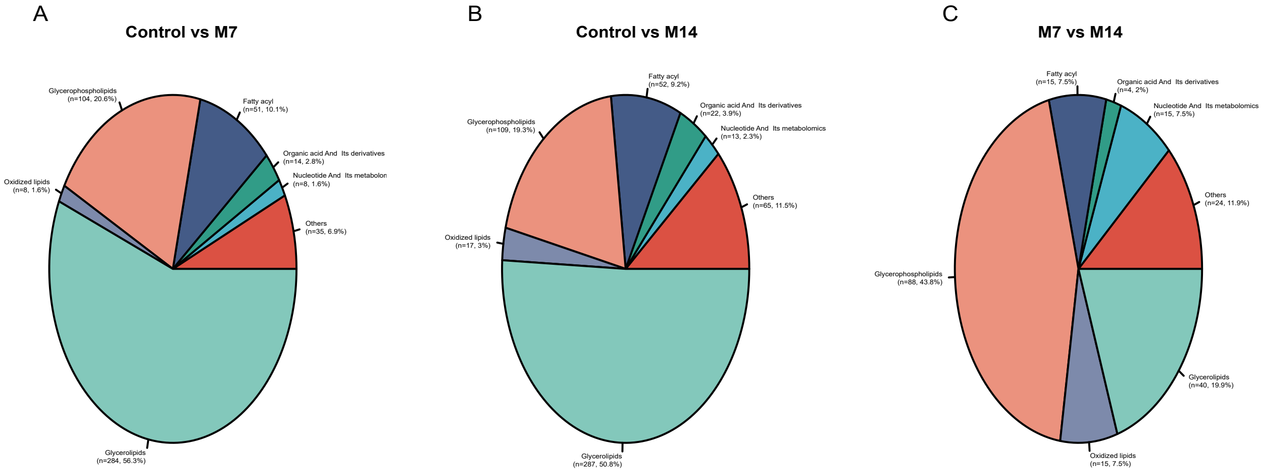

Figure S3 Composition of metabolites differing between groups in the sera of mice with pulmonary fibrosis

(A) Composition of the differential metabolites between Control and M7; (B) Composition of the differential metabolites between Control and M14; (C) Composition of the differential metabolites between M7 and M14.
