# Supplementary material for: Disturbance of serum lipid metabolites and potential biomarkers in the Bleomycin model of pulmonary fibrosis in young mice
Source: BMC Pulm Med. 2022 May 4;22:176. doi: 10.1186/s12890-022-01972-6 (PMC9066762; doi:10.1186/s12890-022-01972-6)
Supplement: Supplementary file 4 — Additional file 4: Original pictures of TGF-β1 and ɑ-SMA expression in mouse lung tissue were analyzed by Western blot at 7 and 14 days after bleomycin perfusion. [file 12890_2022_1972_MOESM4_ESM.pdf]

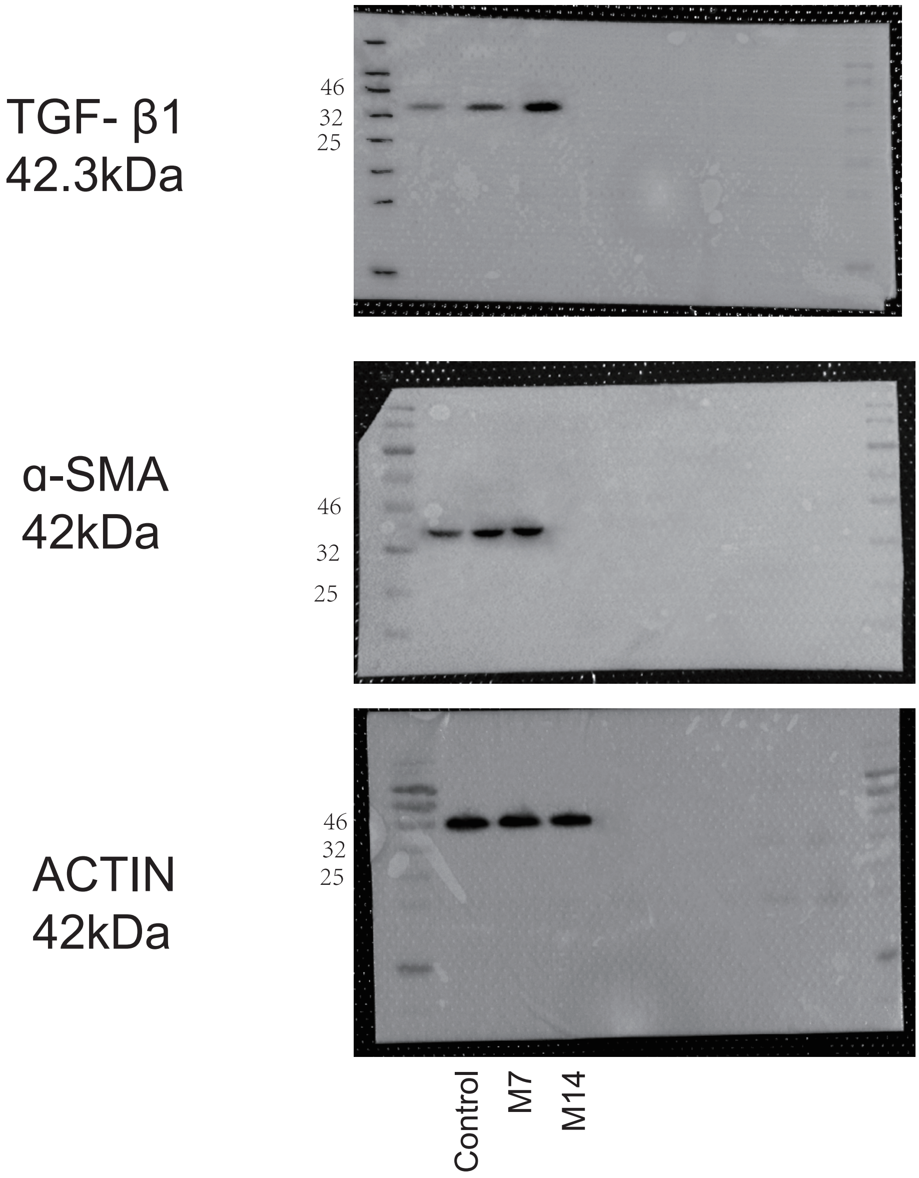

Figure S4 Original pictures of TGF-β and α-SMA expression in mouse lung tissue were analyzed by Western blot at 7 and 14 days after bleomycin perfusion.
